# A new short uncemented, proximally fixed anatomic femoral implant with a prominent lateral flare: design rationals and study design of an international clinical trial

**DOI:** 10.1186/1471-2474-9-147

**Published:** 2008-11-04

**Authors:** Tobias Renkawitz, Francesco S Santori, Joachim Grifka, Carlos Valverde, Michael M Morlock, Ian D Learmonth

**Affiliations:** 1 Regensburg University Medical Center, Bad Abbach, Germany; 2 Ospedale San Pietro Fatebenefratelli, Rome, Italy; 3 University of Bristol, Bristol, UK; 4 Hospital Arnau de Vilanova, Valencia, Spain; 5 Hamburg University of Technology, Hamburg, Germany

## Abstract

**Background:**

Anatomic short femoral prostheses with a prominent lateral flare have the potential to reduce stress-shielding in the femur through a more physiological stress distribution to the proximal femur. We present the design rationale of a new short uncemented, proximally fixed anatomic femoral implant and the study design of a prospective multi-centre trial to collect long-term patient outcome and radiographic follow up data.

**Methods:**

A prospective surveillance study (trial registry NCT00208555) in four European centres (UK, Italy, Spain and Germany) with a follow up period of 15 years will be executed. The recruitment target is 200 subjects, patients between the ages of 18 and 70 admitted for primary cementless unilateral THA will be included. The primary objective is to evaluate the five-year survivorship of the new cementless short stem. The secondary objectives of this investigation are to evaluate the long term survivorship and the clinical performance of the implant, the impact on the subjects health related Quality of Life and the affect of the prosthesis on bone mineral density. Peri- and postoperative complications will be registered. Clinical and radiographic evaluation of prosthesis positioning will be done post-operatively and at 3, 6, 12, 24, 60, 120 and 180 months follow up.

**Discussion:**

Shortening of the distal stem can maximise bone and soft tissue conservation. New stem types have been designed to improve the limitations of traditional implants in primary THA. A new, uncemented femoral short stem is introduced in this paper. A long-term follow up study has been designed to verify stable fixation and to research into the clinical outcome. The results of this trial will be presented as soon as they become available.

## Background

Although substantial progress has been made in the development of cementless total hip arthroplasty (THA) in recent years, a number of limitations remain. The implantation of the femoral component requires a large surface area of bone to be prepared [1]. Osteopenia due to non-physiological loading and stress protection, distal migration of wear particles from the joint space or inadequate stem fixation can increase the risk of aseptic loosening and subsidence of the stem [2-4]. The use of a stiff femoral component may lead to calcar atrophy and cortical thinning, however modern titanium alloy femoral components appear to reduce the risk of this stress-shielding effect [5,6]. Moreover, intense research efforts have been directed at characterising postoperative thigh pain, a clinical limitation in THA that can range from immediate mild postoperative symptoms to severe disabling pain requiring revision surgery [7,8]. Micromotion, loosening, uneven stress patterns or stem tip sclerosis seem to induce such thigh pain [9,10]. Additionally, the use of a long femoral stem increases the risk of thigh pain due to impingement of the stem tip on the femoral cortex [11] and a direct correlation has been drawn between thigh pain and increased stem sizes [12]. Research has also been conducted into ways to improve the limitations of traditional surgical techniques in THA. A less invasive surgical technique may lead to less pain in the early postoperative period and improve the postoperative functional status [13,14] although the scientific discussion about the superior outcomes of this technique compared with the traditional surgical procedure is still ongoing [15,16].

To address such limitations, new THA implant designs with shorter stems have been developed. Some designs, such as the IPS™ [17], the Mayo Conservative Hip or the Santori Custom stem [18,19] have involved shortening or discarding much of the distal stem with the aim of maximising bone and soft tissue conservation. In addition, anatomic short femoral prostheses can reduce the potential for stress-shielding in the femur through a more physiological stress distribution to the proximal femur [20,21]. An uncemented, anatomic, proximally fixed femoral short stem (the DePuy Proxima™ hip, DePuy International, Leeds, UK) has been designed by a team of international surgeons. The purpose of this study is to conduct a prospective clinical trial to collect long-term clinical, patient outcome and radiographic follow up data. Through the long term follow-up, outcomes measures will be compared to conventional cementless femoral components. The present paper reports on the design rationale of this new short stem prosthesis and the methodological design of the study.

### Proximal load transfer

In 1917, John C. Koch [22] proposed his model of the mechanics of the loading of the hip, which included a geometrical description of the femur and a calculation of stresses induced by load that were assumed to occur during gait. By correlating the stress patterns in the trabecular bone with Wolff's [23] concepts of bone formation, Koch assigned compressive and tensile forces along the medial and lateral femoral surfaces. According to his theory, during femoral loading, the superior neck and proximal lateral three quarters of the femoral shaft were under tensile loading while the distal lateral and entire medial femoral surfaces were under compression. Koch's model was considered as the definitive model of hip biomechanics for the next seventy years and served as a basis for the development, design and validation of THA systems. However, as Koch's static model did not sufficiently focus on the function of soft tissues around the hip joint, Fetto et al. [24] published an advanced model in 1995. Through the inclusion of the iliotibial band as a static lateral tension band and the gluteus medius-vastus lateralis complex as dynamic tension bands along the lateral aspect of the lower limb, the authors demonstrated that compressive loading is actually generated both laterally and medially throughout the femur distal to the greater trochanteric apophysis during the unilateral support phase of gait. Further consistency of this model was achieved by bone morphology studies with cadaveric femora and femoral CT scans, revealing a significant amount of cortical bone mass at the lateral aspect of the femur. The authors therefore concluded that the femoral component of THA prostheses should engage the proximal lateral femoral cortex as an additional area of support against subsidence, to avoid stress-shielding and subsequent loss of proximal femoral bone.

### Lateral flare

**Figure 1:**
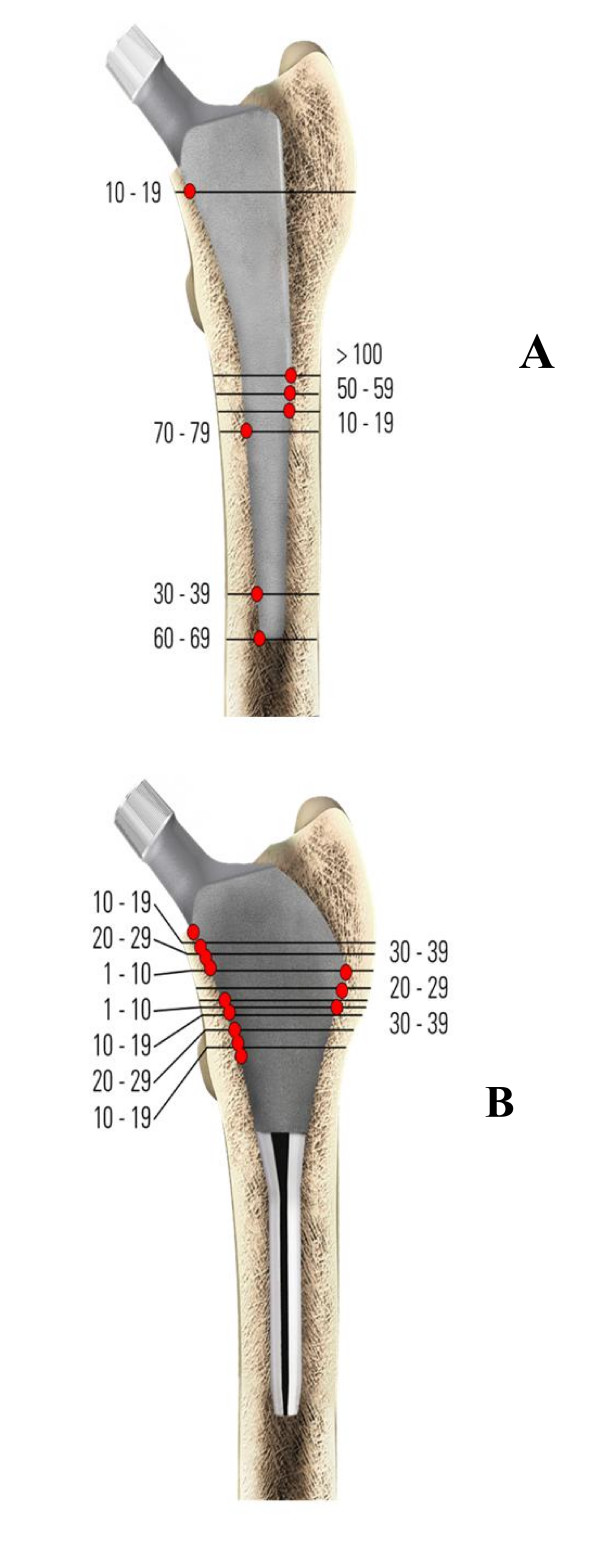
Interface contact stresses (MPA) normal to the surface of a straight stem (1a) and a lateral flare stem (1b), in unbonded conditions, without a collar and with zero interface friction.

Walker at al. [25] discovered fundamental changes in the load transfer between stem and bone when comparing contact pressures between femoral bone and standard length straight stems or stems with a lateral flare. This analysis showed, that for a standard straight stem, loads are mainly transferred through the distal half of the stem (Fig. 1a). In contrast, interface contact stresses from a proximally fixed stem with a lateral flare demonstrated that all of the loading from the prosthesis is transferred to the proximal femur (Fig. 1b). Moreover, the magnitudes of the interface stresses and distal migration during application of the load were both lower in the lateral flare stem. Additionally, results of radiographic follow-up from these authors showed trabecular attachment onto the lateral flare, providing indirect evidence of load transfer in that area. Accordingly, Leali et al. [26] reviewed radiographs from primary THAs with a lateral flare for axial migration and stability. The proximally fixed cementless femoral component showed an average subsidence of 0.32 mm after 2 years, which remained below 1 mm for the duration of the 24–104 month follow-up. The authors concluded that a proximal lateral flare provides significant initial stability, which has been shown to be vital to obtain long-term stability through early bone ingrowth [27]. Additionally, a dual-energy X-ray densitometry study of stems with a lateral flare was performed, which demonstrated that the bone content was preserved at the baseline level or above throughout the follow-up period of 1 year. This was particularly evident in the proximal prosthesis support zones (Gruen zones 1, 2, 6 and 7) [28]. Likewise bone mineral density around the Santori custom short stem was significantly higher in zones 1 and 7 when compared to other conventional cementless implants on a three year follow up [29].

### Product design

**Figure 2:**
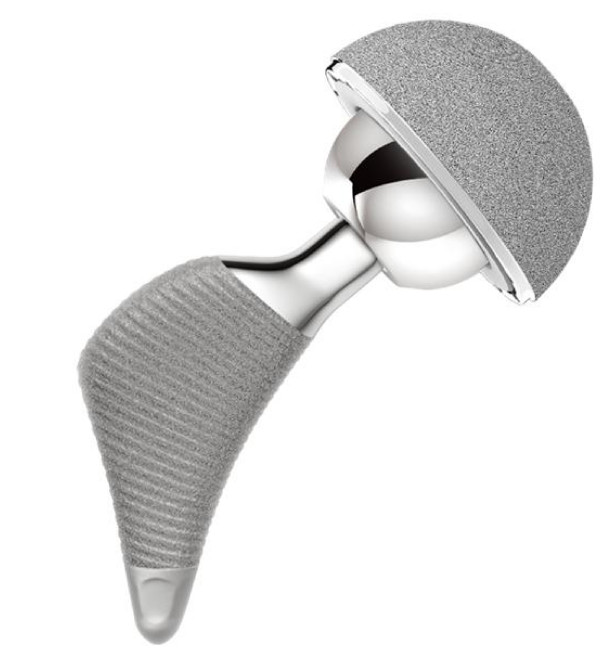
The DePuy Proxima™ hip.

The DePuy Proxima™ hip (DePuy International, Leeds, UK) is manufactured from titanium alloy (Ti-6AL-4V) and has a lateral flare intended to conform to the lateral femoral endosteal surface (Fig. 2). The stem has a 12/14 taper and is available in high and standard offsets. The physiological neck angle is set at 130° and the prosthesis is anatomically shaped with an anteverted neck and available in left and right versions. The entire proximal region of the stem has a sintered bead porous coating with a thin layer of Hydroxyapatite for optimized rapid osseointegration and is stepped to minimise shear forces.

### Surgical technique

Femoral components with a significant lateral flare should be implanted with modified surgical insertion technique, to minimise the potential risk of damage to the greater trochanter [18]. Instruments and implants are therefore first inserted in a slight varus position and then rotated into the correct axial alignment. Adequate cancellous bone for osseointegration should be left on the lateral part of the femur whilst moving distally in the femoral metaphysis. Initially a high neck cut is used, as preservation of the femoral neck has been shown to provide an effective means to achieve immediate, post-operative torsional and coronal stability, which is a key factor in the prevention of loosening of the femoral component of THA [30,31]. Biomechanical stability of the new implant was intensively tested and compared to other clinically successful shaft prostheses prior to the start of the clinical study [32,33].

## Methods

### Study design

This is a multi-centre, prospective, post marketing surveillance study with a follow up period of 15 years (ClinicalTrials.gov Identifier: NCT00208455). It will be open, non-randomised and non-stratified. The primary objective is to evaluate the five-year survivorship of the new cementless short stem. The secondary objectives of this investigation are to evaluate the long-term survivorship and the clinical performance of the implant, the impact on the subjects health related Quality of Life and the affect of the prosthesis on bone mineral density.

### Study population

The study will be conducted in four centres (UK, Italy, Spain and Germany). The recruitment target is 200 subjects, with each centre recruiting 50 subjects. The study has been approved by the ethics committees of Southmead LREC (Bristol, UK), Ospedale San Pietro (Rome, Italy), Hospital Arnau de Vilanova (Valencia, Spain) and University of Regensburg (Regensburg, Germany). Patients included will be aged between 18 and 70 years, suitable for a cementless primary THA and able to understand the study and co-operate with the study follow up visits. The main primary diagnosis will be osteoarthritis. However, other diagnoses will be included such as rheumatoid arthritis, avascular necrosis, developmental dysplasia of the hip or fractures. Excluded patients will be those with other conditions or disorders, unrelated to their hip replacement, that would affect their long-term involvement in the study and patients already participating in other research studies. Only one hip per patient is entered into the study. Patients undergoing a simultaneous bilateral or with a problematic or recent contralateral hip replacement will also be excluded.

### Intervention

Cementless total hip replacements using the DePuy Proxima™ hip component.

### Measurements

#### General measurements

The Harris Hip Score will be used to assess the clinical outcomes. The Oxford Hip Score will be used to assess the patient related outcomes. The effect of the implant on bone mineral density will be assessed using DEXA analysis.

#### Perioperative measurements

Demographic information such as height, weight and age will be collected pre-operatively. In addition information concerning the condition of the operative joint, primary diagnosis, medical history and concomitant medical problems will be collected. The Charnley and DORR classifications will be used to assess the pre-operative joint. Baseline Harris Hip and Oxford Hip scores will also be completed. Intra-operatively, surgical approach, average surgical time, incision length, blood loss and details of any operative complications will be recorded.

#### Radiographic evaluation

**Figure 3:**
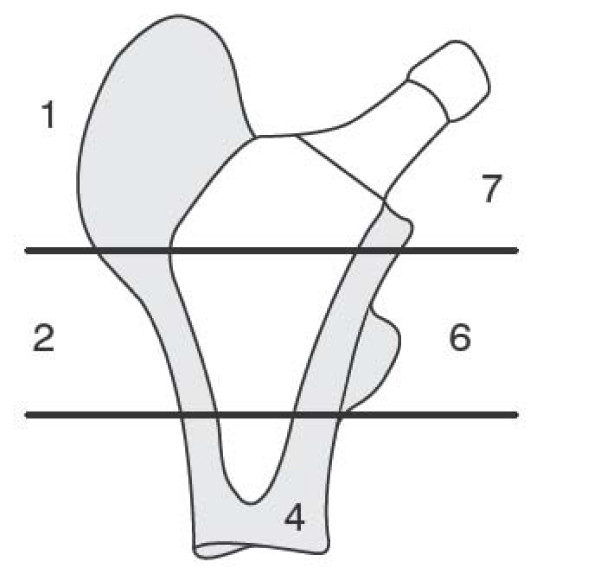
Adapted Gruen zones.

Radiographic evaluations will be done post-operatively and at 3, 6, 12, 24, 60, 120 and 180 months follow up. At each time-point standard AP and Lateral x-rays will be taken and assessed. For the femoral implant signs of radiolucency and osteolysis will be recorded if observed and subsidence will be assessed by recording the position of the component at successive time-points. In addition, evidence of positive bone response will be recorded. Given the lack of a distal portion the Gruen zones have been adapted (Fig. 3). For the acetabular component, evidence of osteolysis or resorption will be recorded, any change in position or orientation will be covered and wear will be measured if possible.

### Statistical analysis

The survivorship will be calculated using both revision and radiological loosening of the implant as end-points for the analysis. Kaplan-Meier survivorship curves will be calculated. Analysis will be performed to assess whether the patient or surgical characteristics have any affect on the primary endpoints. In addition, multivariate analysis using Cox's Proportional Hazards modeling will be undertaken in relation to the survival analysis.

The statistical analysis will be performed by a qualified Biostatistician (Gary Warriner, DePuy International, Leeds, UK) using the statistical package SAS Version 8 (SAS Institute Inc., SAS Campus Drive, Cary, North Carolina 27513) and Microsoft Access. All significance tests will be two-sided and carried out at a 5% significance level, 95% confidence intervals will be regarded as appropriate.

All eligible subjects admitted to the study that receive treatment, and have at least one usable post-treatment assessment, will be included in the statistical analysis where possible. Protocol violators will not be included in the statistical analysis. If applicable, the number of ineligible subjects that received treatment and the reasons for ineligibility will be summarised.

Missing values will be excluded from the analysis as they will be assumed to be missing completely at random and so no loss of information or bias will occur if they are not included in the analysis. If more than 20% of values are missing for a particular analysis variable, then this may significantly bias the results, hence further statistical investigation methods may be employed to try and investigate the effect of missing values.

Subjects who withdraw or are lost to follow-up will be included in the analysis up to the point of withdrawal or last known assessment.

## Discussion

Re-evaluation of the femoral biomechanics by Fetto et al. has shown that the lateral femoral column can be effectively used to carry compressive loads [26,34,35]. Starting from that concept, Santori et al. developed and used a custom-made short stem with a well-defined lateral flare in 111 patients since 1995. Recent clinical and radiographic reviews confirmed the excellent results and positive bone remodelling of these implants [18,19,29]. An uncemented, anatomic, proximally fixed femoral short stem that follows the concept of proximal load transfer through a prominent lateral flare has therefore been developed by an international team of surgeons. Due to the specific shape of the stem an adapted insertion technique is required to avoid damage to the great trochanter and gluteus muscles. The broaching and final implant insertion is performed using a slight curved movement. Biomechanical in vitro tests performed by Westphal et al. with this implant show that adequate stability can be achieved with the selection of a small implant size and cancellous fixation of the stem when good bone quality is present [32,33]. Therefore, a sufficient amount of healthy cancellous bone around the implant should already be considered in the pre-operative templating. To reduce the risk of a varus movement through a higher lever arm, long and extra long femoral heads should not be used with high offset implants. The implant lends itself to minimal invasive surgery done with a Smith-Petersen or MicroHip^® ^approach, where conventional straight stems with a lateral shoulder may increase the risk of damaging the great trochanter and/or the abductor tendons [36,37]. However, one major concern in short hip stems is primary and torsional stability. Whiteside et al. have shown that preserving the femoral neck can effectively reduce micromotion and increase torsional stability [31].

Moreover, Leali et al. reported substantially less migration during loading for lateral flare stems when compared to conventional straight stems [26]. These biomechanical observations confirm, that short stems in general are not ideally suited to all patients. It is important to respect the loading and fixation mechanics when using a metaphyseal implant. This relates to the loading applied to the implant, the surface area available for fixation and the quality of the bone stock. The purpose of the prospective clinical study presented in this article is to collect clinical, patient outcome and radiographic follow up data with a new short stem for primary THA. Long-term follow up is needed to verify stable fixation and continuing successful clinical results with this uncemented, proximally fixed anatomic femoral implant. The results of this study will be presented as soon as they become available.

## Competing interests

TR has received research funding from Depuy International. JG, IDL, CV, MMM and FSC are consultants for Depuy International and have received research funding from Depuy.

## Authors' contributions

All authors participated in the origination for the study, led on its design and will supervise the project. All authors read and corrected draft versions of the manuscript and approved the final manuscript.

## Pre-publication history

The pre-publication history for this paper can be accessed here:


